# Suspension of Anti-VEGF Treatment Does Not Affect Expansion of RPE Atrophy in Neovascular Age-Related Macular Degeneration

**DOI:** 10.3390/jcm12113659

**Published:** 2023-05-25

**Authors:** Shinichiro Chujo, Hisashi Matsubara, Yoshitsugu Matsui, Masahiko Sugimoto, Mineo Kondo

**Affiliations:** Department of Ophthalmology, Mie University Graduate School of Medicine, 2-174 Edobashi, Tsu 514-8507, Japanmineo@med.mie-u.ac.jp (M.K.)

**Keywords:** age-related macular degeneration, AMD, anti-VEGF agent, intravitreal injection, treatment suspension, RPE atrophy

## Abstract

Purpose: To determine whether atrophy of the retinal pigment epithelium (RPE) in eyes with neovascular age-related macular degeneration (nAMD), which meets the criteria for the suspension of anti-vascular endothelial growth factor (anti-VEGF) treatment, is associated with anti-VEGF treatments. Methods: Twelve eyes of 12 patients with nAMD who began anti-VEGF treatment and were followed for 1 year after meeting the criteria for the suspension of anti-VEGF were studied. Six eyes of six patients were placed in the continuation group, and six eyes of six patients were placed in the suspension group. The RPE atrophic area at the time of the last anti-VEGF treatment was set as the baseline size and that at 12 months after the baseline (Month 12) was taken as the final size. A comparison of the expansion rate of RPE atrophy between the two groups was made by the square-root transformed differences. Results: The expansion rate of atrophy was 0.55 (0.43, 0.72) mm/year in the continuation group and 0.33 (0.15, 0.41) mm/year in the suspension group. This difference was not significant. (*p* = 0.29). Conclusions: Suspension of anti-VEGF treatments in eyes with nAMD does not alter the expansion rate of RPE atrophy.

## 1. Introduction

Neovascular age-related macular degeneration (nAMD) is a major retinal disorder that leads to the reduction of vision at an older age. In Japan, the number of patients with nAMD is increasing [1,2,3]. Ranibizumab (Lucentis; Novartis, Brach, Switzerland) is a humanized anti-VEGF antibody fragment designed to bind to all vascular endothelial growth factor (VEGF)-A isoforms [4]. Aflibercept (Eylea; Bayer, Basel, Switzerland) is a soluble decoy receptor fusion protein that consists of the binding domains of the VEGF receptors 1 and 2 fused to the Fc portion of human immunoglobulin G-1. These characteristics allow it to bind to all VEGF-A isoforms as well as VEGF-B and placental growth factor [5]. Several clinical trials on nAMD have shown that monthly or bimonthly injections of ranibizumab or aflibercept were effective in improving and maintaining visual acuity [6,7]. Thus, anti-VEGF agents have been widely used and have become the first-line treatment for nAMD [7]. However, the frequent treatments and hospital visits impose a heavy economic and physical burden on the individuals.

Several methods have been proposed to solve these problems. The treat and extend (TAE) regimen, in which the treatment interval is changed depending on the status of the retinal lesions, is widely used as a treatment regimen. It reduces the burden on patients by decreasing the number of injections and hospital visits while maintaining the improvements in visual acuity [8,9]. To further lessen the burden associated with the treatment protocol, suspension of the anti-VEGF treatment in patients with an absence of disease activity has been performed [10]. However, it has been reported that treatment suspension has a risk of recurrence [11,12,13], leading to irreversible reductions in vision [14,15]. There is also a strong belief that anti-VEGF treatments should not be interrupted [15]. In fact, it has been reported that continual administration of anti-VEGF drugs at fixed intervals resulted in stable or improved vision in 93.2% of eyes with good long-term results over 7 years [16]. Thus, continued anti-VEGF treatment does not reduce the burden associated with the treatments, but it does reduce the risk of reductions in vision due to exudations.

On the other hand, a strong association between nAMD and RPE atrophy was recently reported. The development of and increase in the size of the RPE atrophy have a strong and common risk of reductions in visual function following an initial short-term visual gain [17,18]. In addition, several studies have examined the association between anti-VEGF treatments and RPE atrophy, and they have reported that continued anti-VEGF injections may increase the size of retinal pigment epithelial (RPE) atrophy [19,20,21,22]. If continued anti-VEGF treatment can lead to reductions in vision due to an increase in the size of the RPE atrophy, then continued anti-VEGF treatment is not necessarily beneficial for some patients, even if it decreases the risk of reductions in vision due to exudations. Therefore, determining whether the suspension of anti-VEGF treatment affects the expansion of RPE atrophy should provide new information for deciding whether to continue or discontinue anti-VEGF treatments for eyes with nAMD. Unfortunately, there are no definitive data on whether the continuation or the suspension of anti-VEGF treatment will significantly affect the expansion of RPE atrophy.

Thus, the purpose of this study was to compare the size of the RPE atrophy and the expansion rate of the RPE atrophy between eyes that continued with the anti-VEGF treatment and the eyes that had a suspension of the anti-VEGF treatment.

## 2. Materials and Methods

### 2.1. Study Design

The procedures used in this study were approved by the Ethics Committee of Mie University Hospital (approval number: H2021-088, UMIN000044144), and they conformed to the tenets of the Declaration of Helsinki. The medical records of nAMD patients treated between April 2009 and December 2021 with intravitreal injections of ranibizumab (Lucentis; Novartis, Brach, Switzerland) or aflibercept (Eylea; Bayer, Basel, Switzerland) at the Mie University Hospital (Mie, Japan) were analyzed.

### 2.2. Subjects

The criterion for suspending the anti-VEGF treatments was the absence of exudation or bleeding for at least 48 weeks with continued intravitreal injections of ranibizumab or aflibercept at intervals of 12 weeks or more [11]. The inclusion criteria were: >50 years of age, treated with intravitreal injections of ranibizumab or aflibercept for nAMD for at least 2 years at the Mie University Hospital, treated with the TAE or a fixed-dose regimen before meeting the suspension criteria, and followed for >1 year after meeting the suspension criteria. Patients were excluded if retinal angiomatous proliferation (RAP) or other choroidal neovascularization due to macular disease (e.g., angioid streaks) was present, a refractive error (spherical equivalent) of >−6 diopters, choroidal atrophy due to pathologic myopia was present, prior intraocular surgery within 6 months of the beginning of this study, prior vitrectomy, prior photodynamic therapy or laser photocoagulation, and a history of RPE tear or submacular hemorrhage. Patients who met the criteria for suspension of the anti-VEGF treatment but wished to continue receiving the anti-VEGF treatment were placed in the continuation group, and patients who met the criteria for suspension of the anti-VEGF treatment and who selected to suspend the anti-VEGF treatment were placed in the suspension group.

### 2.3. Quantification of RPE Atrophy

The diagnosis and quantification of RPE atrophy was made by an examination of multimodal images including color photographs of the fundus obtained by a TRC-NW8F retinal camera (Topcon Corp., Tokyo, Japan), blue-light fundus autofluorescence (FAF) images, and optical coherence tomographic (OCT) images obtained by Spectralis^®^ HRA-OCT (HRA2, Heidelberg Engineering, Heidelberg, Germany). The diagnosis of RPE atrophy was made using criteria similar to those used in previous studies. RPE atrophy was identified as lesions within the macular vascular arcade in which the RPE was partially or entirely depigmented in an approximately round or oval-shaped pattern. In addition, the autofluorescence signal in the FAF images was reduced with a thin overlying neurosensory retina in the OCT images. Moreover, the longest linear dimension of the atrophic area was >250 µm with the presence of atrophic changes in the RPE and photoreceptor cells. There was also an increase in the choroidal signals beneath the lesion in the OCT images, and at least one of the following characteristics was present: sharply demarcated borders, underlying choroidal vessels visible, or a uniform RPE atrophic region quantified by the region-finding analyzer (Region Finder Software, Heidelberg Engineering, Heidelberg, Germany) [23].

The size of the RPE atrophic area was quantified at each visit, and the baseline was defined as the administration date of the last anti-VEGF treatment during the period of the suspension criteria. The size on the date of the last visit, which was at least 12 ± 1 months after the baseline visit, was used as the endpoint size. The final determination of the size of the RPE atrophy was made by two retina specialists (SC and HM).

To quantify the RPE atrophic expansion, the expansion rate of the RPE atrophy (mm^2^/year) and the square root of the expansion rate of the RPE atrophy (mm/year) were used. These were used because it has been reported that the expansion rate of RPE atrophy, viz., the increase in expansion per year, reflects the expansion of the RPE atrophy better [23], and it was used in earlier studies [24]. Second, the use of the square root has also been reported to be helpful by excluding the effects of the baseline sites of RPE atrophy [25]. The expansion rate of the RPE atrophy represents the increase in the RPE atrophic area and was calculated by

(Month 12 RPE atrophic area − Baseline RPE atrophic area)/1 year

The expansion rate of atrophy from the baseline to Month 12 was calculated as the square root transformation, and they were statistically compared.

The background factors were those that have been frequently examined as being associated with RPE atrophy in earlier studies [22,23,24]. These included: the subtype of AMD, the presence of classic choroidal neovascularization, the presence of intraretinal fluid at the start of the treatment, the type of anti-VEGF agent used, the number of injections from the start to the baseline, the greatest linear dimension (GLD), and the subfoveal choroidal thickness [22,23,24]. Then, for patients who presented with RPE atrophy at the baseline, their background factors, the size of the RPE atrophy, and the expansion rate of the atrophy were compared between the continuation and suspension groups. For secondary evaluations, a multivariate analysis was performed to determine the association between the expansion of RPE atrophy and the background factors of all eligible patients with RPE atrophy at the baseline. Furthermore, the development of RPE atrophy at Month 12 was evaluated in all eligible patients without RPE atrophy at the baseline.

### 2.4. Statistical Analyses

The descriptive data are presented as numbers, percentages, medians, and first and third quartiles (Q1, Q3) where appropriate. The Mann–Whitney U-test and Fisher’s percentage correct tests were used to compare the background factors between the continuation and suspension groups. The Mann–Whitney U-test was also used to compare the expansion rate of atrophy between the two groups. Multiple regression analyses were performed between the expansion rate of atrophy and the background factors in all eligible patients with RPE atrophy at the baseline. Statistical significance was defined as *p* < 0.05. All statistical analyses were performed using R version 2.9.0.

## 3. Results

### 3.1. Selection of the Participants

**Figure 1:**
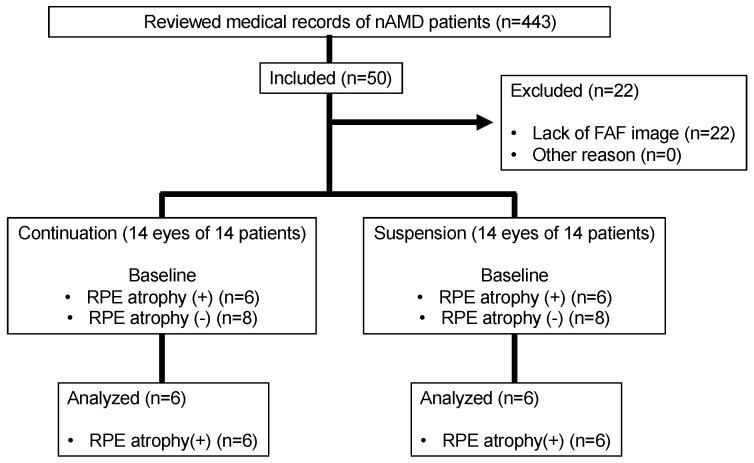
Flow chart showing the selection of the participants. nAMD, neovascular age-related macular degeneration.

**Figure 2:**
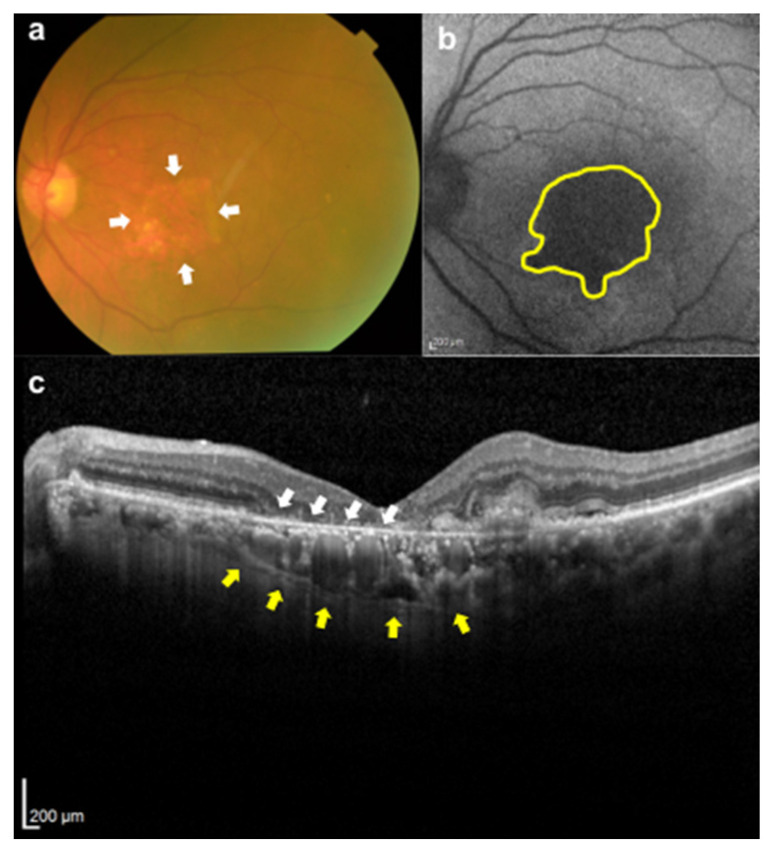
Multimodal images of an 80-year-old man who was receiving anti-VEGF treatment for neovascular age-related macular degeneration. (**a**) Color photograph of the fundus showing RPE atrophy in the macula (white arrows). (**b**) Fundus autofluorescence shows a lesion with a decreased autofluorescence signal corresponding to RPE atrophy (the region enclosed by the yellow line). (**c**) Optical coherence tomography image showing a loss of the outer retinal layer (white arrows) and choroidal hyper-transmission (yellow arrows) corresponding to RPE atrophy. VEGF, vascular endothelial growth factor; RPE, retinal pigment epithelium.

Of the 443 patients whose medical records were examined, 50 met the inclusion criteria. From that group, 22 patients were excluded due to a lack of FAF images. In the end, 14 eyes of 14 patients were included in the continuation and suspension groups. Among these, six eyes in the continuation group and six eyes in the suspension group had RPE atrophy at the baseline (Figure 1). Sixteen patients who met the criteria but did not have RPE atrophy were excluded. Representative cases of RPE atrophy are shown in Figure 2.

### 3.2. Clinical Characteristics of Patients

Eight eyes in the continuation group and eight eyes in the suspension group that had no RPE atrophy at the baseline did not develop RPE atrophy at Month 12. The background factors for each group are shown in Table 1. The differences in the background factors were not statistically significant between the two groups.

### 3.3. Comparisons of RPE Atrophy

**Figure 3:**
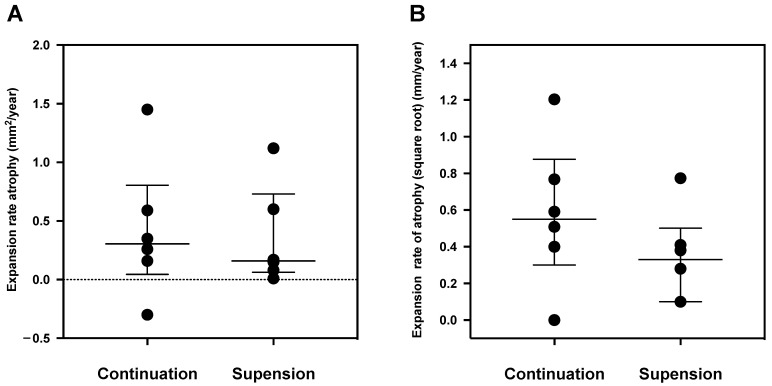
Comparison of the expansion rate of RPE atrophy between the two groups. Bar-dot graph of the expansion rate of RPE atrophy (mm^2^/year) (**A**) and expansion rate of RPE atrophy (square root) (mm/year) (**B**).

The differences in the median (first and third quartiles) area of RPE atrophy and the square root between the continuation and suspension groups are shown in Table 2. At the baseline, the area of RPE atrophy in the continuous group was 2.01 (1.46, 4.42) mm^2^ and that in the suspension group was 0.68 (0.43, 3.05) mm^2^. At Month 12, the area of RPE atrophy was 2.89 (2.05, 4.51) mm^2^ in the continuation group and 0.81 (0.56, 3.6) mm^2^ in the suspension group. The expansion rate of atrophy was 0.55 (0.43, 0.72) mm/year in the continuation group and 0.33 (0.15, 0.41) mm/year in the suspension group (*p* = 0.29; Mann–Whitney U-test). Comparisons of the expansion rate of RPE atrophy between the two groups are shown in bar-dot graphs (Figure 3)

### 3.4. Associations between the Background Factors and the Expansion Rate of RPE Atrophy

In the multivariate analysis between the expansion rate of RPE atrophy and the background factors, there were no factors that were significantly different (Table 3).

## 4. Discussion

Our results showed that there was no significant difference in the rate of expansion of the RPE atrophy between the continuation group and the suspension group. There was also no significant association between the expansion rate of the atrophy and the factors that have been reported to contribute to the expansion of RPE atrophy. Moreover, patients without RPE atrophy at the baseline did not develop RPE atrophy even after 1 year of continued anti-VEGF treatment.

The number of anti-VEGF injections has been cited as a factor that is associated with the progression of RPE atrophy. However, it is not definitively known whether the injection of anti-VEGF drugs is associated with the increase in RPE atrophy. Several recent studies have reported that the number of anti-VEGF injections is associated with the expansion of RPE atrophy [19,20,21,22]. Grunwald reported that a fixed dosing regimen was associated with a greater expansion of RPE atrophy than the PRN regimen in eyes with nAMD. The reason for this is that the VEGF produced by the RPE plays an important role in the maintenance of the choriocapillaris. Therefore, inhibiting VEGF with anti-VEGF drugs may affect the development and progression of RPE atrophy through its action on the choriocapillaris [20]. In contrast, several studies have reported that there was no significant association between the expansion of RPE atrophy and the number of injections of anti-VEGF [23,24]. Casalino et al. [26] reported that in the subset of patients with pre-existing geographic atrophy (GA), the GA significantly increased in both the anti-VEGF-treated and untreated fellow eyes with a similar absolute increase and growth rate. This suggested that there was little causal relationship between the anti-VEGF treatment and the progression of GA.

In earlier studies, the expansion rate of atrophy ranged from 0.31 to 0.57 mm/year in eyes treated with anti-VEGF agents [23,24,26,27], and the expansion rate was 0.21 to 0.58 mm/year without anti-VEGF treatment [28,29,30,31]. In our study, the expansion rate of atrophy was 0.55 mm/year in the continuation group, which is within the range reported for the groups with or without anti-VEGF treatment. The expansion rate of atrophy in the suspension group was 0.33 mm/year, which was not significantly different from that of the continuation group. In addition, the number of anti-VEGF injections was not a significant factor associated with the expansion of RPE atrophy, and eyes without RPE atrophy at the baseline did not develop RPE atrophy whether they continued or suspended the anti-VEGF therapy. These results suggest that the suspension of anti-VEGF does not lead to a decrease in the expansion of RPE atrophy, and that anti-VEGF treatment plays no or little role in the development of RPE atrophy. Furthermore, there was no significant association between the factors that have been reported to be associated with the expansion of RPE atrophy, other than the number of anti-VEGF injections. Therefore, we suggest that factors other than those examined including the anti-VEGF drugs used are associated with the expansion of RPE atrophy. In earlier studies, the minor factors reported to be associated with RPE atrophy included the patients’ genetic predisposition, the shape of the RPE atrophic region, and the presence or absence of complications of RPE atrophy [32,33,34]. Of note, Shmueli and colleagues focused on the shape of the RPE atrophic region and reported that the mechanism by which the RPE cells were exposed at the boundary between the atrophic and normal regions was important. This exposed region accelerated the atrophic processes of apoptosis and immune-related cell death [33]. On the basis of the results of these earlier studies, we suggest that the reasons why there was no significant difference in the expansion of RPE atrophy between the continuation and suspension groups and why RPE atrophy did not develop in patients without RPE atrophy were the following. In patients with RPE atrophy at the baseline, the suspension of anti-VEGF reduced the risk of disruption of the choriocapillaris. However, enhancement of the atrophic process through exposure of the RPE cells persisted. Because a continuation of the anti-VEGF treatment was not a significant factor affecting the expansion of RPE atrophy, the factors that may cause the expansion of RPE atrophy, including the atrophic processes, have not been excluded even if anti-VEGF drug exposure was not present after the treatment was suspended. Therefore, the RPE atrophic region expanded to the same extent in both the continuation and suspension groups.

On the other hand, in cases without RPE atrophy at the baseline, maintenance of the choriocapillaris was inhibited by the continued anti-VEGF treatment, but the RPE cells were not exposed and the atrophic process did not occur. Therefore, no new RPE atrophy developed in a short period of one year. In fact, Adrean and colleagues reported no significant difference in the rate of increase in RPE atrophy during the treatment and after suspension of treatment in the same patients. This suggested that the administration of anti-VEGF may not be the primary cause of the expansion of RPE atrophy, with which our findings are in agreement [35].

According to our results, clinicians involved in AMD treatment should remember the following two points. First, RPE atrophy expands even when anti-VEGF administration is suspended. Therefore, it is necessary to continue FAF and/or other examinations with attention to the expansion of RPE atrophy even after suspension of the anti-VEGF treatment. Second, because there was no significant difference in the expansion of the RPE atrophy between the continued and suspended anti-VEGF agent treatment groups, it is not reasonable to suspend treatment with an anti-VEGF agent to prevent the expansion of RPE atrophy. It has already been reported that there is a high risk of the recurrence of exudation, which can lead to irreversible reductions in vision after suspension. Therefore, the decision to suspend the anti-VEGF treatment should be based on the patient’s wishes and social status, and the status of the fellow eye, and only when there is a sufficient benefit from suspending the treatments.

There are several limitations in this study. The first limitation is the small sample size due to the retrospective nature of this study. Patients were selected strictly according to the previously reported criteria for the suspension of treatment to create a basis for making decisions about whether to discontinue or continue treatment, which was the purpose of this study. As a result, only 50 of 443 nAMD patients met the inclusion criteria at a single institution over the 12-year period. The percentage of patients who met the suspension criteria was not so different from the previously reported rate of 15% who met the suspension criteria in real-world clinical practice [36]. Furthermore, when patients were selected to suspend the treatment after meeting the criteria, they were often transferred to neighborhood clinics by the request by the patient and stopped the follow-up visits to our hospital. In addition, there were many cases of recurrences within the one-year suspension period. Patients who had a recurrence after suspension were excluded according to our selection criteria. As reported, about one-half of the cases had a recurrence within one year of the suspension of treatment [12]. Moreover, 16 of the 28 patients had no RPE atrophy at the baseline. Although these 16 cases provided data on the incidence of RPE atrophy in our study, they did not contribute to the primary outcome. However, the sample size was not large in a previous study of RPE atrophy in anti-VEGF treatment of AMD [35], and real-world clinical studies may also have these limitations.

A second limitation is that the quantification of the area of RPE atrophy by the Region Finder tool was subjective to some degree, and this will have affected the inter-rater error [37]. Arslan et al. emphasized the importance of data quality in addressing such problems [37]. Therefore, to accurately quantify and compare the RPE atrophy area, we strictly excluded cases with poor FAF images or cases that were not examined at the appropriate time. This is another reason for the small sample size in our study.

The third limitation is the relatively short observational period of 1 year, as the follow-up period was 2 years in many previous studies. The short observation period affected the difference in the number of injections between the continuation and suspension groups, and the number of injections may affect the rate of expansion of RPE atrophy. In our study, the follow-up period was initially planned for 2 years. However, few cases were available after 2 years because of the high recurrence rate after discontinuation of the treatment and because patients who had a recurrence were excluded from the follow-up according to our selection criteria. The second reason is that many of the patients who suspended treatment were followed by their local doctors to reduce the burden of hospital visits. Because of these reasons, the number of cases available for a 2-year follow-up at our facility would have been much smaller. This resulted in the evaluation of cases with a 1-year observation period. Longer follow-up periods may lead to larger differences in the number of injections, which may change the results on the expansion rate of RPE atrophy. The small sample size and short observation period are particularly important limitations of this study. Therefore, we plan, in our next project, to evaluate the relationship between suspension or continuation of the treatment and RPE atrophy in a multicenter study with a larger number of patients and a follow-up period of more than 2 years.

## 5. Conclusions

We compared the differences in the expansion rate of RPE atrophy between patients who continued anti-VEGF treatments after meeting the suspension criteria and those who suspended the anti-VEGF treatments. Our results showed no significant difference between the two groups in the expansion rate of RPE atrophy, indicating that RPE atrophy expanded even after the suspension of the anti-VEGF treatment.

Thus, suspension of anti-VEGF treatments in eyes with nAMD does not alter the expansion rate of RPE atrophy. These findings should help clinicians involved in treating AMD in deciding whether to continue or suspend anti-VEGF treatments and manage RPE atrophy after the suspension. Future studies with an increased sample size and extended follow-up periods are needed.

## Figures and Tables

**Table 1:** Comparisons of background factors between the continuation group and suspension group.

Background Factors	Continuation	Suspension	*p*-Value
Sex (female/male)	0/6	4/2	0.06
Age (years)	73 [71, 79.5]	74 [66, 79]	0.92
Subtype (tAMD/PCV)	3/3	5/1	0.54
Classic CNV	1	3	0.24
Intraretinal fluid	1	3	0.54
GLD (μm)	2265.7 [1102, 3092]	1153.2 [799, 2503]	0.90
Subfoveal choroidal thickness (μm)	256.7 [150, 289.5]	210.7 [103, 245.5]	0.12
Number of injections	15.5 [9, 25.5]	12 [12, 18.75]	0.40
Anti-VEGF drugs(ranibizumab/aflibercept)	1/5	1/5	1.00

The numbers in the rows for age, GLD, subfoveal choroidal thickness, and number of injections indicate the median, and the first quartile third quartile (in square brackets). tAMD, typical age-related macular degeneration; PCV, polypoidal choroidal vasculopathy; CNV choroidal neovascularization; GLD, greatest linear dimension; VEGF, vascular endothelial growth factor.

**Table 2:** Comparisons of RPE atrophy between the continuation and suspension groups.

	Continuation	Suspension	*p*-Value
Baseline RPE atrophic area (mm^2^)	2.01 [1.46, 4.42]	0.68 [0.43, 3.05]	0.13
Month 12 RPE atrophic area (mm^2^)	2.89 [2.05, 4.51]	0.81 [0.56, 3.6]	0.13
Expansion rate of atrophy (mm^2^/year)	0.3 [0.18, 0.53]	0.1 [0.09, 0.49]	0.70
Expansion rate of atrophy (square root) (mm/year)	0.55 [0.43, 0.72]	0.33 [0.15, 0.41]	0.29

The numbers in the continuation and suspension columns indicate the median, and the first quartile and third quartile (in square brackets). RPE, retinal pigment epithelium.

**Table 3:** Multivariate analysis of the background factors and the expansion rate of RPE atrophy across subjects.

Background Factors	*p*-Value (Multivariate)
Sex (female or male)	0.30
Age (years)	0.92
Subtype (tAMD or PCV)	0.10
Classic CNV	0.76
Intraretinal fluid	0.07
GLD (μm)	0.35
Subfoveal choroidal thickness	0.46
Number of injections	0.18
Anti-VEGF drugs (ranibizumab or aflibercept)	0.36

RPE, retinal pigment epithelium; tAMD, typical age-related macular degeneration; PCV, polypoidal choroidal vasculopathy; CNV, choroidal neovascularization; GLD, greatest linear dimension; VEGF, vascular endothelial growth factor.

## Data Availability

The datasets used during the current study are available from the corresponding author request.
